# Pentameric ligand-gated ion channels exhibit distinct transmembrane domain archetypes for folding/expression and function

**DOI:** 10.1038/s41598-017-00573-2

**Published:** 2017-03-27

**Authors:** J. P. Daniel Therien, John E. Baenziger

**Affiliations:** Department of Biochemistry, Microbiology, and Immunology University of Ottawa, Ottawa, ON K1H 8M5 Canada

## Abstract

Although transmembrane helix-helix interactions must be strong enough to drive folding, they must still permit the inter-helix movements associated with conformational change. Interactions between the outermost M4 and adjacent M1 and M3 α-helices of pentameric ligand-gated ion channels have been implicated in folding and function. Here, we evaluate the role of different physical interactions at this interface in the function of two prokaryotic homologs, GLIC and ELIC. Strikingly, disruption of most interactions in GLIC lead to either a reduction or a complete loss of expression and/or function, while analogous disruptions in ELIC often lead to gains in function. Structural comparisons suggest that GLIC and ELIC represent distinct transmembrane domain archetypes. One archetype, exemplified by GLIC, the glycine and GABA receptors and the glutamate activated chloride channel, has extensive aromatic contacts that govern M4-M1/M3 interactions and that are essential for expression and function. The other archetype, exemplified by ELIC and both the nicotinic acetylcholine and serotonin receptors, has relatively few aromatic contacts that are detrimental to function. These archetypes likely have evolved different mechanisms to balance the need for strong M4 “binding” to M1/M3 to promote folding/expression, and the need for weaker interactions that allow for greater conformational flexibility.

## Introduction

The folding of transmembrane proteins occurs via two stages^1^. First, independently stable transmembrane α-helices are released into the membrane bilayer by the translocon machinery. Second, independently formed α-helices associate with each other to form higher order structures, which in turn create environments that can drive further insertions of the polypeptide chain and/or promote the binding of prosthetic groups within the nascent protein core^2^. With the muscle-type nicotinic acetylcholine receptor (nAChR), appropriate transmembrane helix-helix associations are also required to mask an endoplasmic reticulum retention motif and thus to signal that the mature receptor is ready to traffic to the cell surface^3^. In the absence of effective helix-helix associations, the immature form remains in the endoplasmic reticulum and is targeted for degradation.

Transmembrane proteins, such as the nAChR and other pentameric ligand-gated ion channels (pLGICs), must strike a balance between strong helix-helix associations that drive folding of the transmembrane domain (TMD) and weaker associations that facilitate inter-helix movements associated with protein conformational change^4, 5^. This balance may also impact on the functional sensitivity of pLGICs to the many allosteric modulators that act on their TMDs^6–12^. For example, the outermost M4 α-helix in the TMD of each nAChR subunit influences channel function^13–19^. M4 likely influences function through its associations with the adjacent transmembrane α-helices, M1 and M3^20, 21^. While effective M4-M1/M3 interactions are required to drive protein folding ultimately leading to expression on the cell surface^22^, intrinsically strong interactions reduce, while weaker interactions enhance functional sensitivity to both the surrounding lipid environment and the potentiating effects of a disease-causing M4 lipid-facing tryptophan substitution^23–25^. In other words, a more malleable M4-M1/M3 interface leads to greater functional sensitivity to altered lipid-protein interactions.

**Figure 1:**
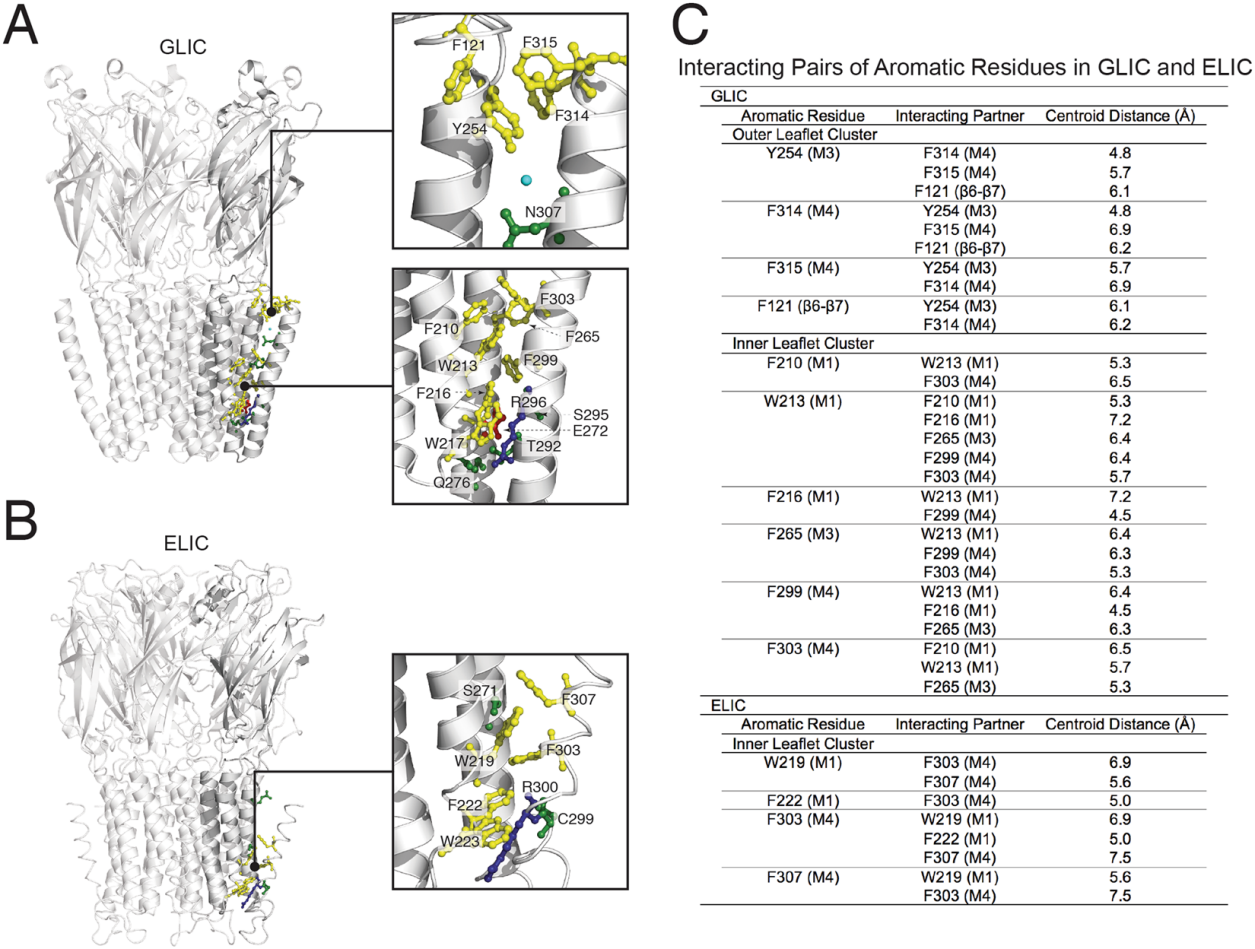
Side chain chemistry at the M4-M1/M3 interfaces of GLIC and ELIC. Structures of (**A**) GLIC (PDB: 4HFI) and (**B**) ELIC (B, PDB: 2VL0) showing on the left the full structure. The boxes on the right show the highlighted regions of GLIC and ELIC. Residues are coloured depending on their properties, aromatic (yellow), hydrogen bonding (green), negatively charged (red) and positively charged (blue). A water molecule in GLIC’s M4-M1/M3 interface is also shown (cyan). Note that aliphatic residues are not shown for clarity. (**C**) The centroid distances between interacting pairs of aromatic residues at the M4-M1/M3 interfaces of GLIC and ELIC.

Aromatic interactions are thought to play a key energetic role driving M4 associations with M1 and M3^22, 23^. Aromatic interactions between M4 and M1/M3 are abundant in GLIC, but less so in ELIC and the nAChR (Fig. 1)^26–28^. In fact, the level of aromatic interactions at this interface varies substantially from one pLGIC subunit to another, leading to the hypothesis that different subunits exhibit different intrinsic strengths of M4-M1/M3 associations and thus different sensitivities to allosteric effectors that act by influencing M4-M1/M3 associations^23, 29^. This hypothesis, however, ignores the possibility that polar and/or van der Waals interactions may compensate for the absence of aromatic residues in some pLGICs to enhance helix-helix associations. The roles of polar and van der Waals interactions at the M4-M1/M3 interface in pLGIC folding and function have not been systematically characterized.

In a previous study, we used Ala scanning mutagenesis to examine the role of M4 in the function of two prokaryotic pLGICs, GLIC and ELIC^29^. Here, we set out to examine more broadly how side chain chemistry at the M4-M1/M3 interface influences GLIC and ELIC function. An unexpected finding of this study, consistent with the findings from the previous M4 Ala scans, is that Ala mutations at the M4-M1/M3 interface in GLIC typically lead to detrimental effects on folding/expression and/or function, while even analogous Ala substitutions in ELIC typically lead to gains in function. M4-M1/M3 interactions appear to be optimized and essential for both expression and function in GLIC, but not in ELIC.

Structural and sequence comparisons suggest that our functional data may reflect the existence of distinct archetypes for the TMDs of pLGICs. One archetype, exemplified by GLIC, the glycine (GlyR), the GABA receptor and the glutamate activated chloride channel (GluCl)^30, 31^, has an M4-M1/M3 interface densely packed with aromatic side chains that form extensive π-π/CH-π interactions to drive M4 associations with M1/M3 during folding^22^. These aromatic interactions, along with a cation-π interaction, an anionic residue located at the core of the M4-M1/M3 interface, and a Pro residue at the midpoint of M4 all facilitate tight, complimentary interface in the folded structure that is essential for function, even though it likely restricts inter-helix motions. In another archetype, exemplified by ELIC, many of the nicotinic receptor subunits, and the serotonin receptor (5HT_3A_R), there are relatively few aromatic side chains at the M4-M1/M3 interface and even fewer inter-helix π-π/CH-π interactions^28, 32^. In fact, the bulky aromatic side chains separate the helices creating gaps at the M4-M1/M3 interface that likely weaken the driving force for M4 associations with M1/M3 during folding, while facilitating inter-helix movements in the folded state. Furthermore, most mammalian members of the latter archetype also lack the cation-π interaction, the anionic residue, and the M4 Pro residue that are essential for a complementary M4-M1/M3 interface in the folded structures of other pLGICs. It appears that these TMD archetypes have evolved different mechanism for balancing the need for strong M4 “binding” to M1/M3 to facilitate folding/expression and the need for weaker interactions that allow for greater conformational flexibility.

## Results

**Figure 2:**
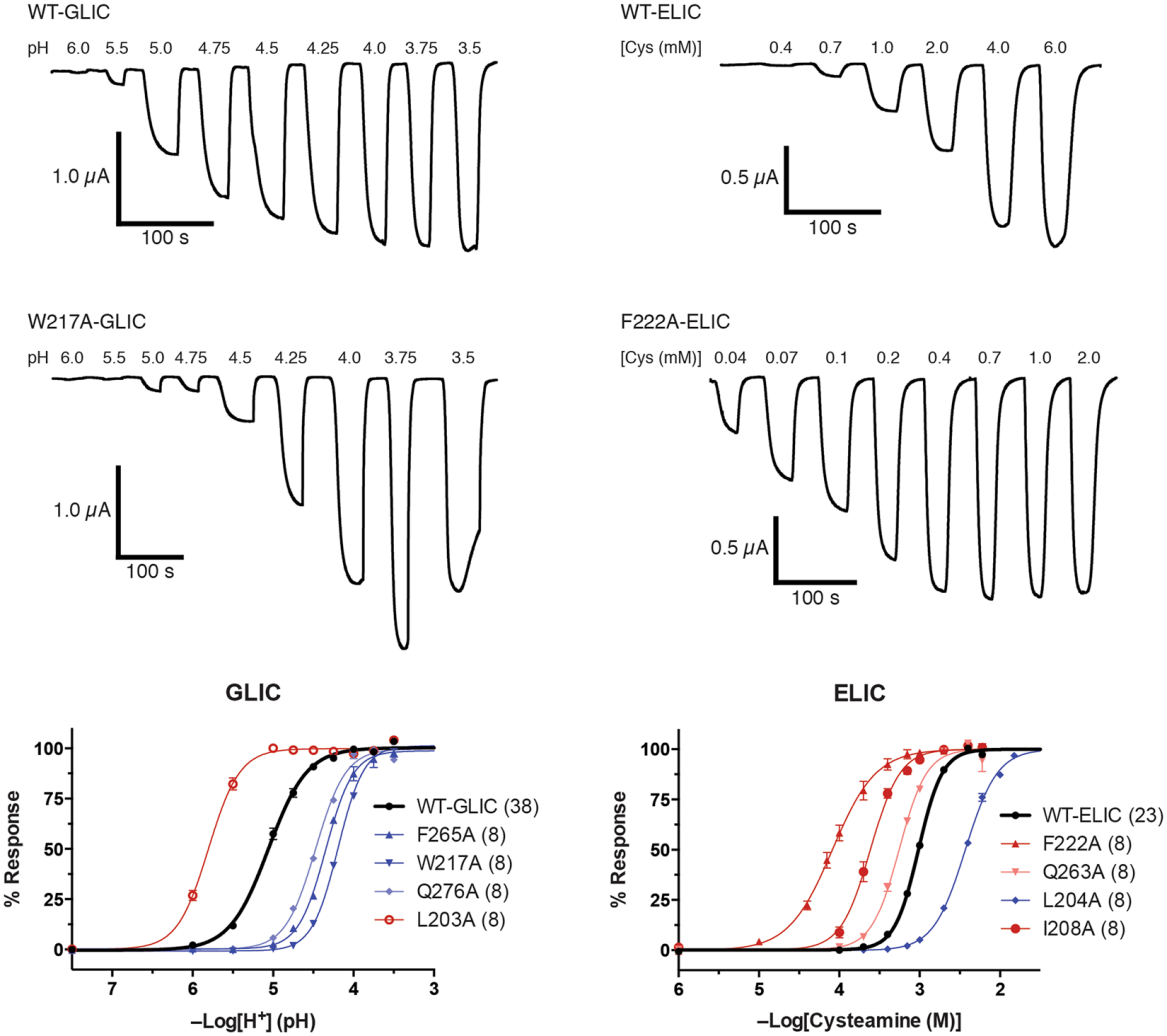
Functional characterization of the GLIC and ELIC mutants. Whole cell electrophysiological traces were recorded using two-electrode voltage clamp electrophysiology. Currents were recorded from *Xenopus laevis* oocytes expressing either GLIC (Left panel) or ELIC (Right panel) in response to protons or cysteamine, respectively. The lower panels presents dose response curves (normalized current (I/I_max_) versus ligand concentration) for select Ala mutants, with the number (n) of averaged traces. Error bars represent S.E.

To examine how different physical interactions at the M4-M1/M3 interface in both GLIC and ELIC (Fig. 1) influence function, we mutated each residue to Ala, and characterized how each mutation influenced agonist-induced channel gating using two-electrode voltage clamp electrophysiology. Both GLIC and ELIC are cation-selective ion channels that transiently gate open in response to agonist binding, although GLIC responds to protons while ELIC responds to primary amines, such as cysteamine^33, 34^. The injection of either wild type (WT) or mutant GLIC/ELIC cRNAs into *Xenopus laevis* oocytes typically led to robust agonist-induced currents across the plasma membrane (Fig. 2), although numerous GLIC mutants and one ELIC mutant did not express and/or function.

**Figure 3:**
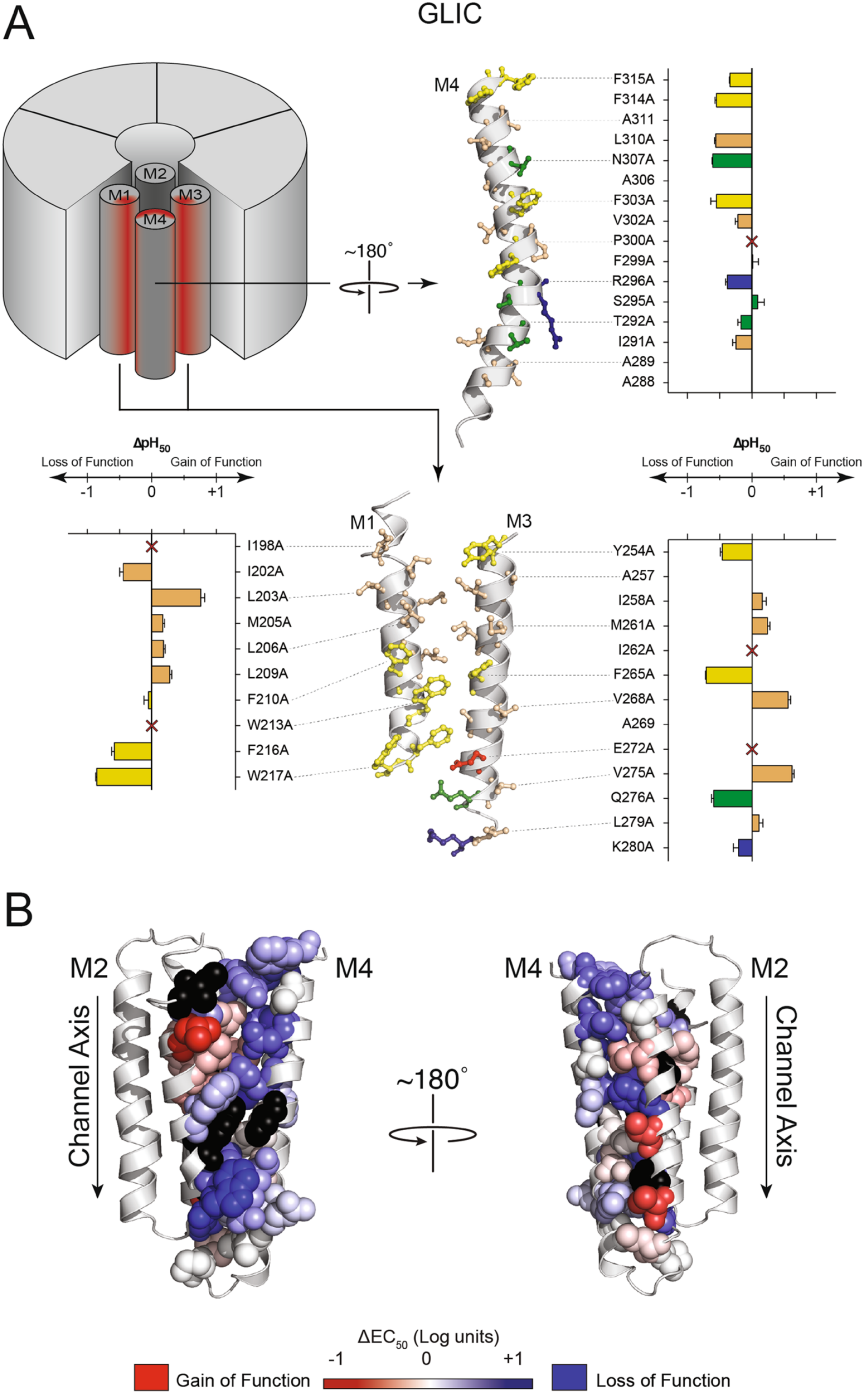
Functional effects of Ala mutations at the M4-M1/M3 interface in GLIC. (**A)** Changes in the pH_50_ resulting from Ala mutations of residues on M4 (top right panel), M1 (bottom left) and M3 (bottom right). Residues and bar graphs are colour-coded as in Fig. 1, with aliphatic residues tan. The bar graphs represent the magnitude of change ± the standard deviation. (**B)** Changes in the pH_50_ values are heat-mapped onto the GLIC structure (PDB: 4HFI). The magnitude of the shift in pH_50_ is depicted via colour intensity, with no change in pH_50_ in white, gain-of-function in red, and loss-of-function in blue. Mutants that failed to express and/or function are shown in black.

**Figure 4:**
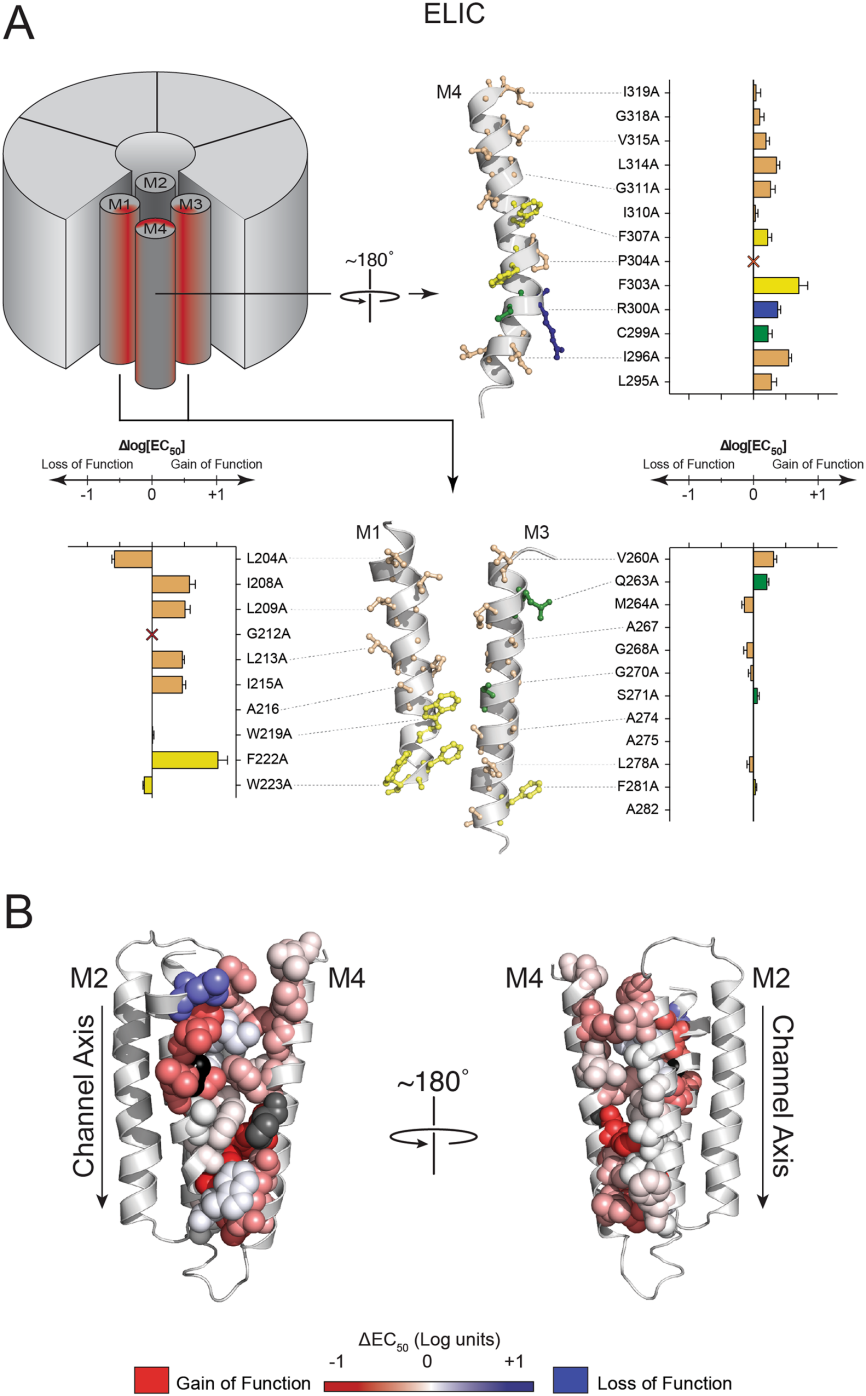
Functional effects of Ala mutations at the M4-M1/M3 interface in ELIC. (**A)** Changes in the −log(EC_50_) resulting from Ala mutations of residues on M4 (top right panel), M1 (bottom left) and M3 (bottom right). Residues and bar graphs are colour-coded as in Fig. 3. The bar graphs represent the magnitude of change ± the standard deviation. (**B)** Changes in the −log(EC_50_) values are heat-mapped onto an ELIC homology model (based on GLIC structure). The magnitude of the shift in pH_50_ is depicted via colour intensity, as in Fig. 3. P304A mutant is coloured grey, due to altered desensitization kinetics.

The Ala-mutations in GLIC and ELIC led to quantitatively similar effects in that all *measured* pH_50_/EC_50_ values were within one full log unit (i.e. ≤10-fold) of WT values. The lack of dramatic changes in channel function is not surprising given the predominantly modest changes in chemistry (36 of 63 mutations are aliphatic to Ala substitutions), and the fact that the mutated side chains are not directly involved in coupling the agonist site to the channel gate^35–37^. The qualitative effects of the mutations, however, were different. Of the twelve Ala mutations in GLIC that led to alterations in the pH_50_ of 0.5 log units or more, nine of these had loss-of-function phenotypes, and an additional five single mutations and most double mutations led to non-functional or non-expressed channels. In contrast, of the eight Ala mutations in ELIC that led to changes in the EC_50_ of 0.5 log units or more, only one had a loss-of-function phenotype, with one additional mutation leading to a non-functional or non-expressed channel and another leading to oocyte cell death. These data show that even subtle changes to the M4-M1/M3 interface in GLIC have detrimental effects on channel function, while similar in ELIC are easily tolerated. It appears that the M4-M1/M3 interface in GLIC is highly optimized for both folding/expression and function, while in ELIC it is not. The measured pH_50_/EC_50_ values for each mutant are grouped according to the chemistry of the affected side chain in Tables 1 and 2. The functional effects of the mutations are mapped onto M1, M3 and M4 in Figs 3 and 4

**Table 1:** The Functional Effects of Alanine Mutations of Residues located at the M4-M1/M3 Interface of GLIC.

GLIC^a^ (WT EC_50_ = 9.33 ± 1.7 µM (pH_50_ = 5.03 ± 0.08) n = 38)							
Single Alanine Mutations							
Aromatic				Aliphatic			
TMD α-helix	Mutant	EC_50_ (pH_50_) (µM)	n	TMD α-helix	Mutant	EC_50_ (pH_50_) (µM)	n
M1	F210A	10.5 ± 1.6 (4.98 ± 0.07)^e^	8	M1	I198A	NC^b^	10
	W213A	NC^b^	17		I202A	25.7 ± 3.3 (4.59 ± 0.06)^e^	8
	F216A	35.5 ± 3.1 (4.45 ± 0.04)^e^	8		L203A	1.6 ± 0.2 (5.79 ± 0.05)^e^	8
	W217A	66.1 ± 3.0 (4.18 ± 0.02)^e^	8		M205A	6.3 ± 0.4 (5.20 ± 0.03)^e^	8
M3	Y254A	26.9 ± 1.8 (4.57 ± 0.03)^e^	8		L206A	6.2 ± 0.4 (5.21 ± 0.03)^e^	8
	F265A	47.9 ± 1.1 (4.32 ± 0.01)^e^	8		L209A	4.9 ± 0.3 (5.31 ± 0.03)^e^	8
M4	F299A	9.1 ± 1.7 (5.04 ± 0.08)^c^	6	M3	I258A	6.5 ± 0.8 (5.19 ± 0.05)^e^	8
	F303A	33.1 ± 6.2 (4.48 ± 0.08)^c,e^	8		M261A	5.4 ± 0.5 (5.27 ± 0.04)^e^	8
	F314A	33.1 ± 1.5 (4.48 ± 0.02)^d,e^	10		I262A	NC^b^	10
	F315A	20.4 ± 0.5 (4.69 0.01)^d,e^	8		V268A	2.6 ± 0.2 (5.59 ± 0.03)^e^	8
Charged					V275A	2.2 ± 0.1 (5.65 ± 0.02)^e^	8
M3	E272A	NC^b^	10		L279A	7.2 ± 0.9 (5.14 ± 0.05)^e^	8
	K280A	15.1 ± 2.5 (4.82 ± 0.07)^e^	8	M4	I291A	16.6 ± 1.8 (4.78 ± 0.05)^c,e^	7
M4	R296A	22.4 ± 1.5 (4.65 ± 0.03)^c,e^	9		P300A	NC^c,b^	8
Hydrogen Bonding					V302A	15.5 ± 1.4 (4.81 ± 0.04)^c,e^	7
M3	Q276A	36.3 ± 3.2 (4.44 ± 0.04)^e^	8		L310A	33.9 ± 1.5 (4.47 ± 0.02)^c,e^	6
M4	T292A	13.8 ± 1.5 (4.86 ± 0.05)^c,e^	6				
	S295A	7.6 ± 1.6 (5.12 ± 0.09)^c,f^	6				
	N307A	38.0 ± 0.9 (4.42 ± 0.01)^c,e^	10				
Double Alanine Mutations							
Outer Leaflet Cluster				Inner Leaflet Cluster			
Mutant		EC_50_ (µM) (pH_50_)	n	Mutant		EC_50_ (µM) (pH_50_)	n
Y254F		7.24 ± 0.8 (5.14 ± 0.05)	8	F210A + F303A		55.0 ± 11.5 (4.26 ± 0.09)	8
Y254F + N307A		27.5 ± 6.4 (4.56 ± 0.10)	8	F216A + F299A		NC^b^	10
Y254A + N307A		40.7 ± 9.4 (4.39 ± 0.10)	8	W217A + R296A		40.7 ± 9.5 (4.39 ± 0.10)	8
Y254A + F314A		NC^b^	12	F265A + F299A		NC^b^	10
Y254A + F315A		NC^b^	11	F265A + F303A		NC^b^	10
F314A + F315A		NC^b^	11	Q276A + T292A		9.55 ± 1.8 (5.02 ± 0.08)	8
				F299A + F303A		NC^b^	10

^a^Data collected 1–4 day(s) after cRNA injection (V_Hold_ ranging from −20 to −40 mV). Error Values represent the standard deviation.

^b^No current (NC) observed in oocytes after 1–4 day(s) of cRNA injection.

^c^Data taken from Henault *et al*.^9, 18, 29^.

^d^Data taken from Carswell *et al*.^23, 24^.

^e^ *p* < 0.001 relative to the pH_50_ of WT GLIC via one-way ANOVA followed by Dunnet’s post hoc test.

^f^ *p* < 0.01 relative to the pH_50_ of WT GLIC via one-way ANOVA followed by Dunnet’s post hoc test.

and are discussed below.

**Table 2:** The Functional Effects of Alanine Mutations of Residues located at the M4-M1/M3 Interface of ELIC.

ELIC^a^ (WT EC_50_ = 0.93 ± 0.13 mM (−Log[EC_50_(M)]) = 3.03 ± 0.06) n = 23)							
Single Alanine Mutations							
Aromatic				Aliphatic			
TMD α-helix	Mutant	EC_50_ (mM) (−logEC_50_)	n	TMD α-helix	Mutant	EC_50_ (mM) (−logEC_50_)	n
M1	W219A	0.91 ± 0.05 (3.04 ± 0.02)	8	M1	L204A	3.55 ± 0.31 (2.45 ± 0.04)^d^	8
	F222A	0.09 ± 0.03 (4.05 ± 0.15)^d^	8		I208A	0.25 ± 0.05 (3.60 ± 0.09)^d^	8
	W223A	1.23 ± 0.06 (2.91 ± 0.02)^d^	8		L209A	0.29 ± 0.05 (3.54 ± 0.08)^d^	8
M3	F281A	0.88 ± 0.05 (3.06 ± 0.02)	8		G212A	NC^b^	10
M4	F303A	0.19 ± 0.07 (3.73 ± 0.17)^c,d^	10		L213A	0.32 ± 0.02 (3.49 ± 0.03)^d^	8
	F307A	0.57 ± 0.09 (3.24 ± 0.07)^c,d^	8		I215A	0.32 ± 0.04 (3.49 ± 0.05)^d^	8
				M3	V260A	0.46 ± 0.05 (3.34 ± 0.05)^d^	8
Charged					M264A	1.29 ± 0.12 (2.89 ± 0.04)^d^	8
M4	R300A	0.40 ± 0.05 (3.40 ± 0.05)^c,d^	11		G268A	1.19 ± 0.14 (2.92 ± 0.05)^d^	8
Hydrogen Bonding					G270A	1.02 ± 0.10 (2.99 ± 0.04)	8
					L278A	1.07 ± 0.10 (2.97 ± 0.04)	8
M3	Q263A	0.58 ± 0.04 (3.24 ± 0.03)^d^	8	M4	L295A	0.50 ± 0.10 (3.30 ± 0.09)^c,d^	8
	S271A	0.82 ± 0.05 (3.09 ± 0.03)	8		I296A	0.27 ± 0.03 (3.57 ± 0.05)^c,d^	10
M4	C299A	0.56 ± 0.08 (3.25 ± 0.06)^c,d^	9		P304A	— ^f^	10
					I310A	0.88 ± 0.09 (3.05 ± 0.04)^c^	10
					G311A	0.51 ± 0.10 (3.29 ± 0.09)^c,d^	11
					L314A	0.42 ± 0.05 (3.38 ± 0.05)^c,d^	8
					V315A	0.60 ± 0.08 (3.22 ± 0.06)^c,d^	9
					G318A	0.76 ± 0.13 (3.12 ± 0.08)^c,e^	10
					I319A	0.86 ± 0.16 (3.06 ± 0.08)^c^	11
Double Alanine Mutations							
Inner Leaflet Cluster				Inner Leaflet Cluster			
Mutant		EC_50_ (mM) (−logEC_50_)	n	Mutant		EC_50_ (mM) (−logEC_50_)	n
W219A + F303A		0.55 ± 0.12 (3.26 ± 0.10)	8	W223A + R300A		0.80 ± 0.11 (3.10 ± 0.05)	8
W219A + F307A		0.45 ± 0.07 (3.35 ± 0.07)	8	S271A + F303A		0.62 ± 0.13 (3.21 ± 0.09)	8
F222A + C299A		0.10 ± 0.02 (4.00 ± 0.09)	8	S271A + F307A		0.77 ± 0.09 (3.11 ± 0.05)	8
F222A + F303A		—^g^	—				

^a^Data collected 1–4 day(s) after cRNA injection (V_Hold_ ranging from −20 to −40 mV). Error Values represent the standard deviation.

^b^No current (NC) observed in oocytes after 1–4 day(s) of cRNA injection.

^c^Data taken from Henault *et al*.^9, 18, 29^.

^d^ *p* < 0.001 relative to the −Log[EC_50_] of WT ELIC via one-way ANOVA followed by Dunnet’s post hoc test.

^e^ *p* < 0.05 relative to the −Log[EC_50_] of WT ELIC via one-way ANOVA followed by Dunnet’s post hoc test.

^f^Functionally distinct, EC_50_ Values were not obtained.

^g^Injected oocytes gave weak response to cysteamine and died past day 1.

### GLIC aromatic residues

The interface between M4 and M1/M3 in GLIC contains a network of ten densely packed and interacting aromatic residues, with one additional aromatic, W217 on M1, involved in a cation-π interaction with R296 on M4. These ten aromatic residues interact with each other via canonical π-π stacking or CH-π interactions that fall within the expected aromatic ring centre-to-centre distances of 4.5 to 7.0 Å (Fig. 1C)^38, 39^. Three interacting aromatics residues located at the M4 C-terminus (F314 and F315) and the M3 N-terminus (Y254) also interact with F121 on the β6-β7 loop^29^ to form the “outer leaflet cluster”. Another seven interacting residues on M1, M3 and M4 are located primarily in the cytoplasmic leaflet of the bilayer (the “inner leaflet cluster”) and form the bulk of the contacts between M4 and M1/M3. A similar network of interacting aromatic residues at the M4-M1/M3 interface of the homologous GlyR plays an essential role in folding/expression and function^22^.

Individual Ala-mutations of eight of the ten aromatic residues led to loss-of-function phenotypes, with one of these leading to a non-expressed and/or non-functional channel (W213) and two mutations (F210A and F299A) having no effect. Of the latter, F210 on M1 interacts only weakly with F303 on M4 (centroid distance of 6.5 Å), while F299 on M4 interacts across the M4-M1/M3 interface with W213 and F216 on M1, and F265 on M3. In the latter case, the cation-π interaction between the adjacent R296 on M4 and W217 on M1 may stabilize functionally important M1-M4 interactions, despite the F299A mutation. Note that W217A led to the largest measurable loss-of-function of any of the aromatic-to-Ala substitutions.

While the detrimental effects of the individual aromatic-to-Ala mutations demonstrate that aromatic residues at the M4-M1/M3 interface are important, a critical role for these residues in folding/expression and function was revealed when two interacting aromatic residues were simultaneously mutated to Ala (Table 1). Ala substitutions of any two of the four side chains in the extracellular aromatic cluster led to a complete loss of folding/expression and/or function. The simultaneous Ala substitution of two interacting residues in the cytoplasmic aromatic cluster was also not tolerated in four of five cases. The one double mutant that still functioned involves F210 on M1 and F303 on M4. As noted, the F210A mutation had essentially no effect. The double F210A/F303A mutant was slightly more detrimental to function than the individual F303A mutant (pH_50_ = 4.26 vs 4.48, respectively).

### GLIC Aliphatic residues

Through van der Waals interactions, aliphatic residues play an important role in transmembrane helix-helix associations^40, 41^. In GLIC, Ala mutations of aliphatic residues at the M4-M1/M3 interface led to the largest variability in their effects on function, with some leading to gains in function, others to losses in function, and most having no effect. Many of the Ala mutations that led to changes in function, however, involve residues that face the M4-M1/M3 interface, but that are not directly involved in M4-M1/M3 contacts. For example, M4-facing residues on the N-terminal half of M1 (I198, I202, L203, M205 and L206) do not directly interact with M4 (Fig. 1A). Two residues on M3, V268 and V275 extend from M3 towards M4, but their contacts with residues on M4 are minimal - they likely interact more extensively with lipids. I262 extends from M3 towards M1 and M4, but is also close to a His residue on M2, H235, which has been implicated in channel function^42, 43^, possibly explaining why the I262A mutant led to a loss of expression and/or function. Many of the changes in function observed with the Ala mutations of aliphatic residues thus likely occur via mechanisms that do not directly involve altered M4-M1/M3 interactions.

A few of the aliphatic mutations that alter EC_50_ values are notable. L209A, which led to a modest gain-of-function, is located in a pocket formed by three interacting residues, F210 and W213 on M1 and F303 on M4 in the inner leaflet aromatic cluster. The smaller Ala side chain may allow greater conformational flexibility to promote more effect interactions between F210/W213 and F303. On the other hand, L310A, which led to a modest loss of function, is located just below the outer leaflet cluster of aromatic residues. In this case, the bulky Leu side chain may fill a void between M4 and M3 created by the adjacent bulky aromatic side chains. Finally, the Ala-mutation of a proline residue on M4 (P300A) led to a complete loss of folding/expression and/or function. P300 kinks M4 so that the C-terminus projects towards the N-terminus of M3. P300A should eliminate this kink and thus dramatically alter how M4 positions itself relative to M1 and M3. This mutation highlights the critical importance of M4-M1/M3 interactions in folding/expression and/or function. P300 may also interact favourably with the W213 on M1^44^.

### GLIC polar residues

Polar residues are key drivers of helix-helix interactions in the hydrophobic membrane environment, but are less important within the interiors of polytopic membrane proteins^4, 5, 45^. GLIC exhibits a number of both charged and neutral polar residues at the M4-M1/M3 interface. Notably, E272 is surrounded by three polar hydrogen bonding residues (Q276, S295, and T292) and likely forms a weak hydrogen bond with T292 (3.1 Å from proton donor to acceptor). E272A did not express and/or conduct cations in response to protons, possibly due to a critical role solvating these three polar groups. On the other hand, individual mutations of any of these three hydrogen bonding groups had minimal effects, with the largest being a modest loss-of-function with the Q276A mutant. Even simultaneous Ala substitutions of the two closest hydrogen bonding residues, Q276A/T292A, had no effect on activity. Interactions with other residues thus likely satisfy the electrostatic requirements of E272. The E276A mutation highlights the importance of charged residues in the folding/expression and/or function of pLGICs, particularly if they are located at the core of interacting TMD α-helices. An anionic residue at this position is conserved in the GlyR, the GABA receptor and in GluCl.

Two positively charged residues are also located at this interface, although K280 near the C-terminus of M3 has no potential interacting polar residues on M4 and likely projects into the bilayer to interact with the polar lipid head groups. The K280A mutant had no effect on channel function. Conversely, R296 on M4 forms a cation-π interaction with W217 on M1. The R296A mutation leads to a modest loss-of-function (pH_50_ = 4.65), while the W217A mutation leads to the largest quantifiable loss-of-function (~10-fold) observed in this study. The double R296A/W217A mutation has a similar effect (pH_50_ = 4.39), suggesting that these two residues are energetically coupled and that this coupling promotes GLIC function.

Finally, Y254 at the N-terminus of M3 not only interacts with adjacent aromatic residues, but its hydroxyl group also forms a hydrogen bond with N307 on M4 through an intervening water molecule^27^. Y254 was mutated to both Phe and Ala, and N307 to Ala. All three mutations had modest detrimental effects on channel function. Both the Y254F/N307A and Y254A/N307A double mutants had EC_50_ values similar to the EC_50_ values of the single mutants. Y254 and N307 are thus energetically coupled, with the interaction having a positive effect on channel function.

### ELIC aromatic residues

ELIC contains only four of the ten interacting aromatic residues found at the M4-M1/M3 interface in GLIC, as well as a Trp residue (W223) on M1 involved in an analogous cation-π interaction with R300 on M4. Notably absent are the three aromatic residues that in GLIC form the extracellular aromatic cluster. Surprisingly, while the aromatic residues in ELIC share homologous positions with those found in GLIC, Ala substitutions had either little effect or actually led to gain of function phenotypes. In fact, the F222A mutation led to a 10-fold gain-of-function while the same mutation in GLIC (F216A) led to a loss-of-function. Even more surprising, double Ala mutations of interacting aromatic residues had no detrimental effect on folding/expression and/or function (Table 2). Even the W223A mutation, which eliminates the cation-π interaction with R300, had close to WT activity. In contrast to GLIC, aromatic residues at the M4-M1/M3 interface in ELIC negatively impact on channel function.

### ELIC Aliphatic residues

Ala mutations of aliphatic residues at the M4-M1/M3 interface in ELIC led to 0.5 log unit changes in EC_50_ in only six of eighteen cases, with only one of these leading to a modest loss-of-function (Table 2). The smaller Ala side chains may allow for closer inter-helix packing to enhance channel function. The smaller side chains could also create space that allows greater conformational flexibility to promote conformational change. Notably, the one aliphatic-to-Ala mutation, L204A, that led to a loss of function is located on M1, but does not interact directly with M4. The corresponding I198A mutation in GLIC led to a complete loss of folding/expression and/or function. In addition, G212A, which is also located in the N-terminal half of M1, did not yield agonist-induced currents. Flexibility at this site appears to be essential for folding/expression and/or function. Finally, P304A on M4 led to channels with substantially altered desensitization kinetics, which made it impossible to accurately predict the effective EC_50_. The analogous P300A mutation in GLIC is not functional.

### ELIC polar residues

ELIC has only one charged residue, R300 on M4, which forms a cation-π interaction with W223 on M1. As noted, the W223A mutant had little effect on channel function. R300A and the W223A/R300A double mutant both led to slight gain-of-function phenotypes suggesting that, unlike in GLIC, this cation-π interaction does not promote channel gating. ELIC also has three hydrogen bonding side chains at the M4-M1/M3 interface, with Q263 and S271 located on M1, and C299 located on M4. None of these residues are close enough to form hydrogen bonds with other polar residues, although S271 and C299 are both close to adjacent aromatic side chains. Mutation of any of these residues alone or in combination with potential interacting partners either led to no change or a decrease in EC_50_, indicating no or gain-of-function phenotypes, respectively.

## Discussion

We set out to understand the roles of different physical interactions at the M4-M1/M3 interfaces of GLIC and ELIC in channel function. Our work was performed in the context of previous findings, which showed that enhanced M4-M1/M3 interactions in GLIC/ELIC promote, while weakened interactions inhibit function^23^. The intrinsic strengths of M4-M1/M3 interactions also influence the functional sensitivities of GLIC/ELIC to both lipids and a lipid-facing M4 Trp mutation^23–25^, which in the muscle nAChR leads to a congenital myasthenic syndrome^13^. Given the variable side chain chemistry at the M4-M1/M3 interfaces of different pLGIC subunits, these preliminary findings suggested that different pLGIC channel subunits exhibit different intrinsic strengths of M4-M1/M3 interactions, possibly leading to different sensitivities to allosteric modulators that act via the M4-M1/M3 interface.

In single transmembrane peptides, van der Waals contacts and polar interactions play a major role driving helix-helix associations. Inter-helix contacts are often mediated by a leucine-zipper like motif (“small-xxx-small”, where small refers to Ala, Gly or Ser)^46, 47^. Leucine zipper motifs facilitate tight interactions between adjacent helices leading to extensive van der Waals contacts. In some cases, these tight interactions even promote the formation of inter-helix hydrogen bonds involving the Cα hydrogen of glycine^45^. Polar interactions are also common driving forces for helix-helix associations, particularly with interacting transmembrane peptides immersed in the hydrophobic bilayer environment^5^. Polar side chain mutations and non-polar to polar mutations can lead to altered or inappropriate interactions that lead to human disease^5, 48–50^. In complex polytopic membrane proteins, however, the tight packing of α-helices is not as prevalent given the increased number of α-helices in the TMD and the necessity to allow for the conformational flexibility required for protein function. Hydrogen bonding interactions in polytopic membrane proteins are typically weaker than expected for a hydrophobic transmembrane environment^4, 51, 52^.

Our mutagenesis data are consistent with findings from other polytopic membrane proteins and suggest that both van der Waals and polar interactions do not play as important a role in TMD helix-helix associations as in single transmembrane peptides. Leucine zipper-like motifs do not govern interactions between M4 and M1/M3 in either GLIC or ELIC and thus do not mediate helix-helix interactions during folding/expression or function. Ala substitutions of aliphatic side chains typically had little effect, suggesting that close van der Waals contacts at the M4-M1/M3 interactions are not essential to GLIC or ELIC function. Similarly, most Ala mutations of polar residues at the M4-M1/M3 interfaces of GLIC and ELIC had modest or no effects, with two notable exceptions. A cation-π interaction between M4 and M1 enhances function in GLIC, but is detrimental to function in ELIC. In addition, a glutamate residue surrounded by polar residues at the core of M4-M1/M3 interactions in GLIC is critical to folding/expression and/or function. A homologous anionic side chain is lacking in ELIC, but found in other pLGICs (see below).

Instead, interactions between M4 and M1/M3 are dominated in both GLIC and ELIC by bulky aromatic residues, although a key finding of this study is that aromatic side chains have diametrically opposing roles in the two pLGICs. In GLIC, individual aromatic to Ala substitutions at the M4-M1/M3 interface typically lead to loss of function phenotypes, with double mutations of interacting aromatic side chains almost invariably leading to a complete loss of folding/expression and/or function. In contrast, both individual and double aromatic-to-Ala mutations at the M4-M1/M3 interface in ELIC typically have little effect or lead to gains in function. Aromatic residues at the M4-M1/M3 interface are thus essential for both folding/expression and function in GLIC. Surprisingly, aromatic residues at the M4-M1/M3 interface are detrimental to function in ELIC. Note that aromatic interactions typically contribute between −0.6 and −1.3 kcalmol^−1^ to the stability of a protein structure^38^. Although direct measurements are required to define the energetic contributions of aromatic residues to M4-M1/M3 interactions, the aromatic residues at the M4-M1/M3 interface of GLIC should contribute substantial free energy to promote M4-M1/M3 associations during both folding/expression and function. Indeed, the thermal denaturation temperature of GLIC is higher than in ELIC^24^.

A comparison between the GLIC and ELIC structures provides a rationale for the distinct phenotypes observed with the Ala mutations of aromatic and other residues. In GLIC, ten aromatic residues at the M4-M1/M3 interface form a network of bulky interacting side chains, with an additional aromatic side chain involved in a cation-π interaction between M1 and M4 (Fig. 1). There is also a glutamate residue at the core of the M4-M1/M3 interface that is solvated by adjacent polar residues. These and other side chains lead to a densely packed and complementary interface along the entire length of M4 that appears to be highly optimized for function, with even subtle modifications to this interface having detrimental effects. In contrast, there are only four aromatic residues at the M4-M1/M3 interface in ELIC, and these make fewer contacts with other aromatic side chains than analogous aromatic side chains in GLIC (Fig. 1). While these bulky aromatic side chains located in the inner leaflet of the bilayer should promote helix-helix associations, the absence of bulky aromatic side chains in the outer leaflet of the bilayer leads to gaps between the transmembrane helices. In fact, the final five residues in M4 are unresolved in ELIC crystal structures suggesting relatively weak interactions between the C-terminal half of M4 and M1/M3. The simplest interpretation of our ELIC mutagenesis data is that in the absence of an extensive network of interacting aromatic residues along the entire length of M4, the bulky aromatic side chains that are present actually prevent the optimal helix-helix associations that are required for channel function^23^. In ELIC, Ala mutations of aromatic side chains may reduce steric barriers and/or lead to greater conformational flexibility to promote optimal M4-M1/M3 interactions.

**Figure 5:**
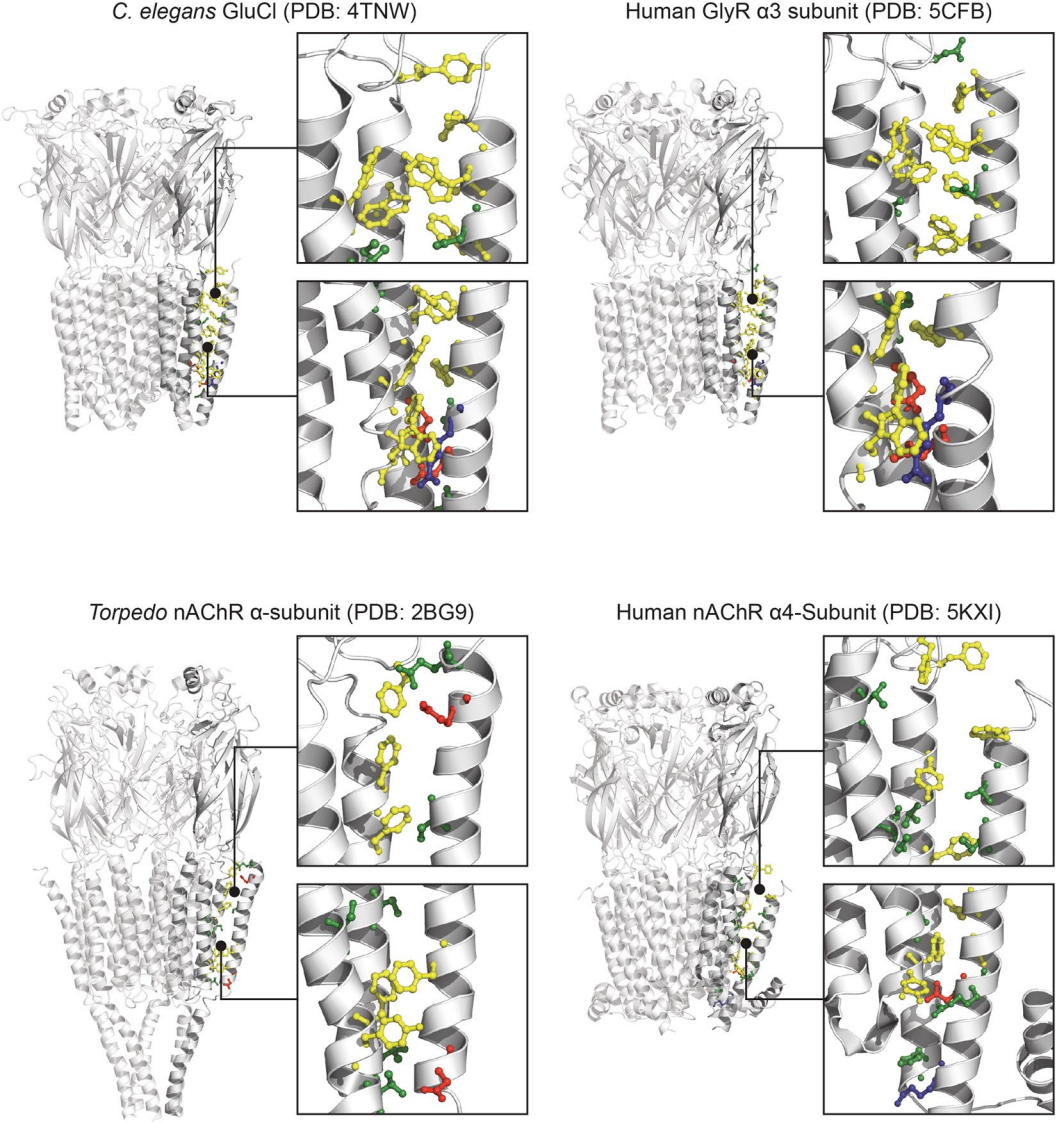
The M4-M1/M3 interface of different pLGICs. Structures of GluCl (top left, PDB: 4TNW), GlyR (top right, PDB: 5CFB), nAChR (bottom left, PDB: 2BG9), and α4β2 (bottom right, PDB: 5KXI). Each structure shows a zoomed in region of the M4-M1/M3 interface of a single subunit. Residues are coloured depending on their properties: aromatic (yellow), hydrogen bonding (green), negatively charged (red) and positively charged (blue).

In this context, it is interesting to note that many neuronal pLGICs have side chain chemistries at the M4-M1/M3 interface similar to GLIC. The GlyR, the GABA receptor and the GluCl each has an extensive network of interacting aromatic residues that leads to a densely packed M4-M1/M3 interface along the entire length of M4 (Fig. 5). Also similar to GLIC, Ala mutations of residues at the M4-M1/M3 interface in the GlyR lead predominantly to loss-of-function phenotypes, with several leading to a complete loss of folding/expression and/or function^22^. As in GLIC, strong interactions at this interface in these pLGICs likely promote folding leading to cell surface expression^22^, although tight “binding” of M4 in the folded structure may hinder inter-helix motions and thus conformational rearrangements of the TMD α-helices during channel gating and desensitization. An intriguing question to address is how this TMD archetype has evolved to gate open despite the conformational restrictions that may be imposed by the strongly interacting residues at the complementary interface between M4 and M1/M3.

In contrast, the M4-M1/M3 interface side chain chemistry in ELIC resembles that of the various subunits of the nAChR and the 5HT_3A_ receptor in they all have relatively few interacting aromatic side chains at the M4-M1/M3 interface. Based on our data, we would predict that the bulky aromatic side chains at this interface actually prevent close helix-helix associations leading to weaker M4 “binding” to M1/M3 in the folded state. This prediction is consistent with the observation that functional receptors are still observed when the hydrophobic transmembrane portion of M4 in the α-subunit of the *Torpedo* nAChR is replaced by the hydrophobic transmembrane segments of either the vesicular stomatitis virus glycoprotein or the human interleukin-2 receptor^53^. Preliminary studies in our lab show that aromatic to Ala substitutions at this interface in the human muscle-type α-subunit lead to gains of function (data not shown). The lack of an extensive aromatic network at the M4-M1/M3 interface in ELIC, the nAChR and the 5HT_3A_ receptor may weaken M4-M1/M3 associations thus facilitating inter-helix movements during conformational change. On the other hand, the absence of an extensive network may weaken the driving force behind M4 “binding” to M1/M3 during folding/expression, thus possibly hindering pLGIC assembly. It is intriguing to ask how these pLGICs have evolved to fold/express in the absence of an extensive aromatic network at the M4-M1/M3 interface - particularly given that even subtle perturbations to the extensive aromatic networks in GLIC and the GlyR often lead to a complete loss of folding/expression and/or function^22^.

**Figure 6:**
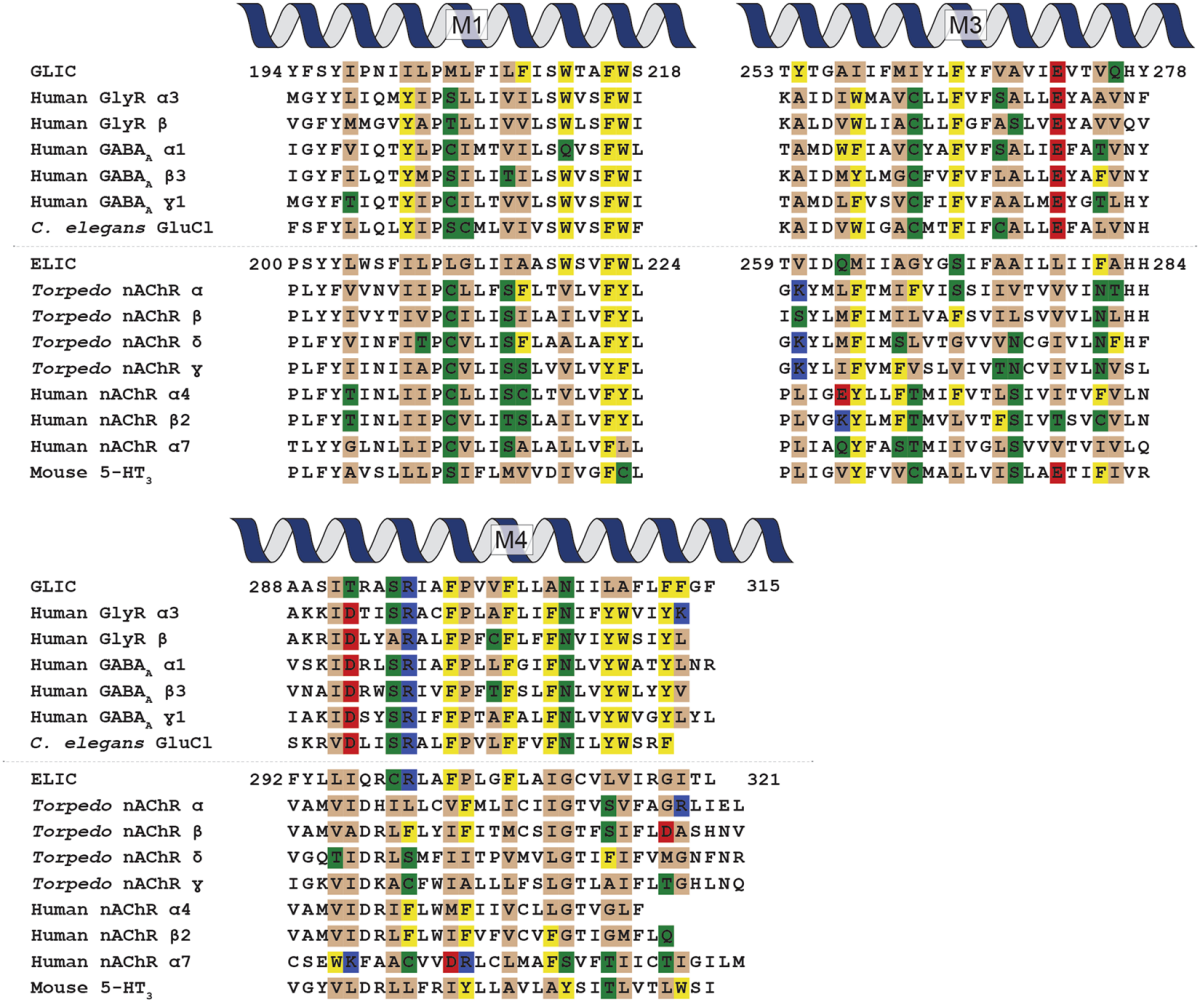
Sequence alignments for M1, M3 and M4 in a number of pLGICs. Residues facing the M4-M1/M3 interface are highlighted and colored depending on their properties: aromatic (yellow), hydrogen bonding (green), negatively charged (red) and positively charged (blue).

In addition to distinct levels of aromatic residues at the M4-M1/M3 interface, the two TMD archetypes share other conserved features (Fig. 6). GLIC, the GlyR and the GABA receptor and GluCl all contain a Pro residue in M4 that kinks the helix to maximize contacts between the C-terminal half of M4 and the N-terminal half of M3. They all have a conserved cation-π interaction linking M1 to M4. Each also has a Glu residue on M3 that is located at the core of M4-M1/M3 interactions. These residues in GLIC and the GlyR all play an important role, in some cases being essential for folding/expression and/or function. In contrast, the various nAChR subunits and the 5-HT_3A_ receptor typically lack the M4 Pro residue, the cation-π interaction linking M1 to M4, and the central Glu residue located on M3. Note that although ELIC fits into the latter archetype based on the level of aromatic residues at the M4-M1/M3 interface, it is unique in that it does contain both the M4 Pro and the cation-π interaction between M1 and M4. ELIC thus exhibits features characteristic of both archetypes.

Our data adds to an increasing literature describing studies where structure-function relationships have been compared and contrasted in GLIC and ELIC^29, 54–57^. Some of these reports, including this study, highlight differences in sequence and function between GLIC and ELIC. The reported findings have raised the possibility that ELIC represents a distinct branch in the pLGIC family^29, 54^. Other reports highlight conserved mechanistic features^57^. Further studies with these two model pLGICs should continue to provide a wealth of information regarding the mechanisms underlying pLGIC function.

## Materials and Methods

### RNA constructs for oocyte expression

The GLIC and ELIC coding sequences, preceded by an α7 nAChR signal sequence, were cloned into plasmids pSP64 and pTLN, respectively. The two plasmids were linearized by EcoRI and MluI, respectively. The linearized plasmids were then used to produce capped cRNA by *in vitro* transcription using the mMESSAGE mMACHINE® SP6 kit (Ambion). All mutants were created using QuikChange™ Site Directed Mutagenesis kits (Agilent) and were verified by sequencing.

### Electrophysiology

Stage V–VI oocytes were collected as previously described^58^, injected with cRNA, and allowed to incubate one to four days at 16 °C in ND96 + buffer (5 mM HEPES, 96 mM NaCl, 2 mM KCl, 1 mM MgCl_2_, 1 mM CaCl_2_, and 2 mM Pyruvate). Whole cell currents were recorded using a two-electrode voltage clamp apparatus (OC-725C oocyte clamp; Holliston, MA). For GLIC, whole cell currents were recorded from injected oocytes (3–20 ng cRNA) immersed in MES buffer (140 mM NaCl, 2.8 mM KCl, 2 mM MgCl_2_, and 10 mM MES). Currents through the plasma membrane in response to pH jumps (pH 7.3 down to the indicated pH values) were measured with the transmembrane voltage clamped at voltages between −20 mV and −40 mV depending on the level of expression, typically −20 mV. For ELIC, whole cell currents were recorded from injected oocytes (0.1–10 ng cRNA) immersed in HEPES buffer (150 mM NaCl, 0.5 mM BaCl_2_, and 10 mM HEPES, pH 7.0). In most cases, currents through the plasma membrane in response to cysteamine concentration jumps (from 0 mM up to the indicated values) were measured with the transmembrane voltage clamped at −30 mV.

Dose responses for each mutant were acquired from at least two different batches of oocytes. Each individual dose-response experiment was fit with a variable slope sigmoidal dose-response and the individual EC_50_ values from each experiment averaged to give the reported EC_50_ values ± standard deviation.

## References

[1] Popot JL, Engelman DM (1990). Membrane protein folding and oligomerization: the two-stage model. Biochemistry.

[2] Engelman DM (2003). Membrane protein folding: beyond the two stage model. FEBS Lett.

[3] Wang JM (2002). A transmembrane motif governs the surface trafficking of nicotinic acetylcholine receptors. Nat Neurosci.

[4] Joh NH (2008). Modest stabilization by most hydrogen-bonded side-chain interactions in membrane proteins. Nature.

[5] Partridge AW, Therien AG, Deber CM (2002). Polar mutations in membrane proteins as a biophysical basis for disease. Biopolymers.

[6] Nury H (2011). X-ray structures of general anaesthetics bound to a pentameric ligand-gated ion channel. Nature.

[7] Forman SA, Chiara DC, Miller KW (2015). Anesthetics target interfacial transmembrane sites in nicotinic acetylcholine receptors. Neuropharmacology.

[8] daCosta CJB, Free CR, Corradi J, Bouzat C, Sine SM (2011). Single-channel and structural foundations of neuronal alpha7 acetylcholine receptor potentiation. J Neurosci.

[9] Baenziger JE, Henault CM, Therien JP, Sun J (2015). Nicotinic acetylcholine receptor-lipid interactions: Mechanistic insight and biological function. Biochim Biophys Acta.

[10] Barrantes FJ (2015). Phylogenetic conservation of protein-lipid motifs in pentameric ligand-gated ion channels. Biochim Biophys Acta.

[11] daCosta CJB (2009). Anionic lipids allosterically modulate multiple nicotinic acetylcholine receptor conformational equilibria. J Biol Chem.

[12] Baenziger JE, Morris ML, Darsaut TE, Ryan SE (2000). Effect of membrane lipid composition on the conformational equilibria of the nicotinic acetylcholine receptor. J Biol Chem.

[13] Shen XM, Deymeer F, Sine SM, Engel AG (2006). Slow-channel mutation in acetylcholine receptor alphaM4 domain and its efficient knockdown. Ann Neurol.

[14] Lee YH (1994). Mutations in the M4 domain of Torpedo californica acetylcholine receptor dramatically alter ion channel function. Biophys J.

[15] Lasalde JA (1996). Tryptophan substitutions at the lipid-exposed transmembrane segment M4 of Torpedo californica acetylcholine receptor govern channel gating. Biochemistry.

[16] Bouzat C, Roccamo AM, Garbus I, Barrantes FJ (1998). Mutations at lipid-exposed residues of the acetylcholine receptor affect its gating kinetics. Mol Pharmacol.

[17] Bouzat C, Barrantes F, Sine S (2000). Nicotinic receptor fourth transmembrane domain: hydrogen bonding by conserved threonine contributes to channel gating kinetics. J Gen Physiol.

[18] Henault CM (2015). The role of the M4 lipid-sensor in the folding, trafficking, and allosteric modulation of nicotinic acetylcholine receptors. Neuropharmacology.

[19] Barrantes FJ (2003). Modulation of nicotinic acetylcholine receptor function through the outer and middle rings of transmembrane domains. Curr Opin Drug Discov Devel.

[20] daCosta CJB, Baenziger JE (2009). A lipid-dependent uncoupled conformation of the acetylcholine receptor. J Biol Chem.

[21] daCosta CJB, Dey L, Therien JP, Baenziger JE (2013). A distinct mechanism for activating uncoupled nicotinic acetylcholine receptors. Nat Chem Biol.

[22] Haeger S (2010). An intramembrane aromatic network determines pentameric assembly of Cys-loop receptors. Nat Struct Mol Biol.

[23] Carswell CL (2015). Role of the Fourth Transmembrane a Helix in the Allosteric Modulation of Pentameric Ligand-Gated Ion Channels. Structure.

[24] Carswell CL, Sun J, Baenziger JE (2015). Intramembrane Aromatic Interactions Influence the Lipid Sensitivities of Pentameric Ligand-gated Ion Channels. J Biol Chem.

[25] Labriola JM (2013). Structural sensitivity of a prokaryotic pentameric ligand-gated ion channel to its membrane environment. J Biol Chem.

[26] Hilf RJ, Dutzler R (2008). X-ray structure of a prokaryotic pentameric ligand-gated ion channel. Nature.

[27] Sauguet L (2013). Structural basis for ion permeation mechanism in pentameric ligand-gated ion channels. EMBO J.

[28] Unwin N (2005). Refined structure of the nicotinic acetylcholine receptor at 4Å resolution. J Mol Biol.

[29] Henault CM, Juranka PF, Baenziger JE (2015). The M4 Transmembrane a-Helix Contributes Differently to Both the Maturation and Function of Two Prokaryotic Pentameric Ligand-gated Ion Channels. J Biol Chem.

[30] Du J, Lu W, Wu S, Cheng Y, Gouaux E (2015). Glycine receptor mechanism elucidated by electron cryo-microscopy. Nature.

[31] Althoff T, Hibbs RE, Banerjee S, Gouaux E (2014). X-ray structures of GluCl in apo states reveal a gating mechanism of Cys-loop receptors. Nature.

[32] Morales-Perez, C. L., Noviello, C. M. & Hibbs, R. E. X-ray structure of the human a4b2 nicotinic receptor. *Nature* (2016). 10.1038/nature19785 PMC5161573 27698419

[33] Bocquet N (2007). A prokaryotic proton-gated ion channel from the nicotinic acetylcholine receptor family. Nature.

[34] Zimmermann I, Dutzler R (2011). Ligand activation of the prokaryotic pentameric ligand-gated ion channel ELIC. PLoS Biol.

[35] Lee WY, Sine SM (2005). Principal pathway coupling agonist binding to channel gating in nicotinic receptors. Nature.

[36] Grutter T (2005). Molecular tuning of fast gating in pentameric ligand-gated ion channels. Proc Natl Acad Sci USA.

[37] Lummis SC (2005). Cis-trans isomerization at a proline opens the pore of a neurotransmitter-gated ion channel. Nature.

[38] Burley SK, Petsko GA (1985). Aromatic-aromatic interaction: a mechanism of protein structure stabilization. Science.

[39] Waters ML (2002). Aromatic interactions in model systems. Curr Opin Chem Biol.

[40] Russ WP, Engelman DM (2000). The GxxxG motif: a framework for transmembrane helix-helix association. J Mol Biol.

[41] Rath A, Johnson RM, Deber CM (2007). Peptides as transmembrane segments: decrypting the determinants for helix-helix interactions in membrane proteins. Biopolymers.

[42] Rienzo M, Lummis SC, Dougherty DA (2014). Structural requirements in the transmembrane domain of GLIC revealed by incorporation of noncanonical histidine analogs. Chem Biol.

[43] Wang HL, Cheng X, Sine SM (2012). Intramembrane proton binding site linked to activation of bacterial pentameric ion channel. J Biol Chem.

[44] Biedermannova L, Riley KE, Berka K, Hobza P, Vondrasek J (2008). Another role of proline: stabilization interactions in proteins and protein complexes concerning proline and tryptophane. Phys Chem Chem Phys.

[45] Bowie JU (2011). Membrane protein folding: how important are hydrogen bonds?. Curr Opin Struct Biol.

[46] Kleiger G, Grothe R, Mallick P, Eisenberg D (2002). GXXXG and AXXXA: common alpha-helical interaction motifs in proteins, particularly in extremophiles. Biochemistry.

[47] Curran AR, Engelman DM (2003). Sequence motifs, polar interactions and conformational changes in helical membrane proteins. Curr Opin Struct Biol.

[48] Partridge AW, Melnyk RA, Deber CM (2002). Polar residues in membrane domains of proteins: molecular basis for helix-helix association in a mutant CFTR transmembrane segment. Biochemistry.

[49] Smith SO, Smith CS, Bormann BJ (1996). Strong hydrogen bonding interactions involving a buried glutamic acid in the transmembrane sequence of the neu/erbB-2 receptor. Nat Struct Biol.

[50] Weiner DB, Liu J, Cohen JA, Williams WV, Greene MI (1989). A point mutation in the neu oncogene mimics ligand induction of receptor aggregation. Nature.

[51] Zhou FX, Cocco MJ, Russ WP, Brunger AT, Engelman DM (2000). Interhelical hydrogen bonding drives strong interactions in membrane proteins. Nat Struct Biol.

[52] Choma C, Gratkowski H, Lear JD, DeGrado WF (2000). Asparagine-mediated self-association of a model transmembrane helix. Nat Struct Biol.

[53] Tobimatsu T (1987). Effects of substitution of putative transmembrane segments on nicotinic acetylcholine receptor function. FEBS Lett.

[54] Gonzalez-Gutierrez G, Grosman C (2015). The atypical cation-conduction and gating properties of ELIC underscore the marked functional versatility of the pentameric ligand-gated ion-channel fold. J Gen Physiol.

[55] Schmandt N (2015). A chimeric prokaryotic pentameric ligand-gated channel reveals distinct pathways of activation. J Gen Physiol.

[56] Henault CM, Baenziger JE (2017). Functional characterization of two prokaryotic pentameric ligand-gated ion channel chimeras - role of the GLIC transmembrane domain in proton sensing. Biochim Biophys Acta.

[57] Bertozzi C, Zimmermann I, Engeler S, Hilf RJ, Dutzler R (2016). Signal Transduction at the Domain Interface of Prokaryotic Pentameric Ligand-Gated Ion Channels. PLoS Biol.

[58] Laitko U, Juranka PF, Morris CE (2006). Membrane stretch slows the concerted step prior to opening in a Kv channel. J Gen Physiol.

